# Comparative study of equine mesenchymal stem cells from healthy and injured synovial tissues: an in vitro assessment

**DOI:** 10.1186/s13287-016-0294-3

**Published:** 2016-03-05

**Authors:** Joice Fülber, Durvanei A. Maria, Luis Cláudio Lopes Correia da Silva, Cristina O. Massoco, Fernanda Agreste, Raquel Y. Arantes Baccarin

**Affiliations:** Department of Internal Medicine, School of Veterinary Medicine and Animal Science, University of São Paulo (USP), Avenida Prof. Orlando Marques de Paiva, 87, 05508-270 São Paulo, SP Brazil; Laboratory of Biochemistry and Biophysics, Butantan Institute, Avenida Vital Brasil 1500, São Paulo, 05503-900 SP Brazil; Department of Surgery, School of Veterinary Medicine and Animal Science, University of São Paulo (USP), Avenida Prof. Orlando Marques de Paiva, 87, SP, 05508-270 SP Brazil; Department of Pathology, School of Veterinary Medicine and Animal Science, University of São Paulo (USP), Avenida Prof. Orlando Marques de Paiva, 87, São Paulo, 05508-270 SP Brazil

**Keywords:** Equine, Mesenchymal stem cell, Synovial fluid, Synovial membrane, Allogeneic cell bank

## Abstract

**Background:**

Bone marrow and adipose tissues are known sources of mesenchymal stem cells (MSCs) in horses; however, synovial tissues might be a promising alternative. The aim of this study was to evaluate phenotypic characteristics and differentiation potential of equine MSCs from synovial fluid (SF) and synovial membrane (SM) of healthy joints (SF-H and SM-H), joints with osteoarthritis (SF-OA and SM-OA) and joints with osteochondritis dissecans (SF-OCD and SM-OCD) to determine the most suitable synovial source for an allogeneic therapy cell bank.

**Methods:**

Expression of the markers CD90, CD105, CD44, and CD34 in SF-H, SM-H, SF-OA, SM-OA, SF-OCD and SM-OCD was verified by flow cytometry, and expression of cytokeratin, vimentin, PGP 9.5, PCNA, lysozyme, nanog, and Oct4 was verified by immunocytochemistry. MSCs were cultured and evaluated for their chondrogenic, osteogenic and adipogenic differentiation potential. Final quantification of extracellular matrix and mineralized matrix was determined using AxioVision software. A tumorigenicity test was conducted in Balb-C^nu/nu^ mice to verify the safety of the MSCs from these sources.

**Results:**

Cultured cells from SF and SM exhibited fibroblastoid morphology and the ability to adhere to plastic. The time elapsed between primary culture and the third passage was approximately 73 days for SF-H, 89 days for SF-OCD, 60 days for SF-OA, 68 days for SM-H, 57 days for SM-OCD and 54 days for SM-OA. The doubling time for SF-OCD was higher than that for other cells at the first passage (P < 0.05). MSCs from synovial tissues showed positive expression of the markers CD90, CD44, lysozyme, PGP 9.5, PCNA and vimentin and were able to differentiate into chondrogenic (21 days) and osteogenic (21 days) lineages, and, although poorly, into adipogenic lineages (14 days). The areas staining positive for extracellular matrix in the SF-H and SM-H groups were larger than those in the SF-OA and SM-OA groups (P < 0.05). The positive mineralized matrix area in the SF-H group was larger than those in all the other groups (P < 0.05). The studied cells exhibited no tumorigenic effects.

**Conclusions:**

SF and SM are viable sources of equine MSCs. All sources studied provide suitable MSCs for an allogeneic therapy cell bank; nevertheless, MSCs from healthy joints may be preferable for cell banking purposes because they exhibit better chondrogenic differentiation capacity.

## Background

Osteoarticular diseases have received a substantial amount of scientific attention in recent years, primarily because of their high prevalence and significant impact on the equine industry. These diseases irreparably damage articular cartilage and negatively influence athletic performance in horses. Proper treatment has therefore been sought to facilitate the regeneration of hyaline cartilage and to maintain the integrity of its structure. For this purpose, the use of cellular therapies, including mesenchymal stem cells (MSCs) from various sources, is a promising tool for the treatment of osteoarticular disease.

MSCs are characterized by their proliferative ability and their capacity to differentiate into several mesenchymal lineages, such as osteoblasts, chondrocytes, adipocytes, tenocytes, and myocytes; therefore, they are classified as multipotent progenitor cells [1–3]. Regenerative medicine provides an opportunity to control the evolution of the disease due to the immunomodulatory, anti-inflammatory, and tissue regenerative properties of MSCs. In this context, the use of appropriate populations appears to be crucial for the successful regeneration of damaged articular structures [4].

Regarding horses, MSCs have been obtained from bone marrow, adipose tissue [5], umbilical cord [6–8], umbilical cord blood [9], amniotic membrane [10], peripheral blood [11], and recently from synovial fluid (SF) and synovial membrane (SM) [12, 13].

Although MSCs from synovial tissues have abilities comparable with those of MSCs from other sources, they have also been shown to possess high chondrogenic potential. Additionally, it was inferred that these cells are already predisposed to differentiate into chondrocytes, suggesting that the ancestral microenvironment directs the “destination” cell upon differentiation [14]. These observations support the hypothesis that these cells may be prime candidates for the regeneration of cartilage [15–18]. SM collection can be performed during arthroscopy [4, 19–22], and SF can easily be collected through arthrocentesis [23].

Although autologous therapy with MSCs does not result in any deleterious effects, its use in horses still has limitations, such as the inability to initiate treatment immediately after arthroscopic diagnosis because the expansion of MSCs in culture takes 15–26 days [3]. The treatment of older horses is also limited because there is an apparent decrease in the abilities of MSCs in this population. Allogeneic treatment eliminates the long timeframe that is required to isolate and expand MSCs.

Typically, SF or SM is harvested in cases of osteoarthritis (OA) or osteochondritis dissecans (OCD) during an arthroscopic procedure that is being conducted for prognostic purposes, and these samples can also be used to create a cell bank for allogeneic therapy. However, it is not currently known whether these cell sources exhibit characteristics similar to those of cells from healthy joint tissues.

Even in the case of allogeneic therapy, the harvest of synovial tissue during arthroscopic treatments of joints with OA or OCD may not the best choice, as SF or SM could instead be harvested from contralateral healthy joints. However, there have been few reports of the biological characterization of equine MSCs from synovial tissues, so concomitant quantitative and qualitative assessment should therefore be encouraged to identify these synovial-derived cells and to provide additional information about them.

Based on this research scenario, we outlined a study to compare the phenotype, morphology, and multilineage differential potential of MSCs from synovial fluid (SF-MSCs) and from synovial membrane (SM-MSCs) of horses, using healthy joints, joints with OA, and joints with OCD. Further, to verify that these SF-MSCs and SM-MSCs would not differentiate into tumoral cells, we used a mouse tumorigenicity test.

## Methods

### Animal ethics

This study was conducted in accordance with the Ethics Committee on the Use of Animals of the School of Veterinary Medicine and Animal Science of the University of São Paulo; the protocol number was 2871/2013.

### Collection of SF and SM

In this study, a total of 97 joints from 68 horses were examined. The horses ranged in age from 2 to 10 years, and no restrictions were placed on breed, sex, or joint. SF and SM of healthy joints (SF = 14; SM = 16), of joints with OCD (SF = 21; SM = 16), and of joints with OA (SF = 16; SM = 11) were collected and used in this experiment.

Horses with joint diseases were examined at the Veterinary Hospital of the School of Veterinary Medicine and Animal Science of the University of São Paulo (FMVZ/USP), and arthroscopic surgery was indicated for use as their treatment. In the surgical center, SF and SM samples were collected from joints with OCD and from joints with OA. Immediately prior to the procedure, SF samples were obtained by arthrocentesis using 40 × 10 hypodermic needles. SM samples were collected during surgery using conventional arthroscopic forceps.

Samples of SF and SM from healthy joints were collected at the beginning of the arthroscopic procedures in concurrent experiments.

### Isolation and culture of SF-derived cells

First, 2 ml of harvested synovial fluid from healthy joints (SF-H), from joints with OCD (SF-OCD), and from joints with OA (SF-OA) was suspended in 3 ml of Dulbecco’s modified Eagle medium (DMEM; LGC Biotechnology, Cotia, São Paulo, Brasil) and 10 % (v/v) fetal bovine serum (FBS; Life Technologies, São Paulo, SP, Brasil) supplemented with 1 % penicillin/streptomycin, 1 % glutamine (200 mM), 1 % pyruvic acid, and 0.25 % amphotericin B (Life Technologies, São Paulo, SP, Brasil) and then plated in a 25 cm^2^ flask at a cell density of 3.5 × 10^2^/ml (SF-H), 1.91 × 10^6^/ml (SF-OCD), or 1.93 × 10^6^/ml (SF-OA). Next, the samples were allowed to attach during incubation at 37 °C in a humidified atmosphere containing 5 % CO_2_. On the fourth day, the medium was aspirated to remove nonadherent cells and was replaced with fresh medium. The cell cultures were maintained for sufficient time to monitor cell growth via inverted microscopy, and fresh medium was provided every 48 hours until the cells reached 80 % confluence.

### Isolation and culture of SM-derived cells

Harvested synovial membrane from healthy joints (SM-H), from joints with OCD (SM-OCD), and from joints with OA (SM-OA) were washed with phosphate-buffered saline (PBS) containing 1 % penicillin under sterile conditions to remove debris and blood. Next, approximately 400 mg of SM were gently debrided with a sterile syringe plunger and distributed in a 25 cm^2^ flask with 200 μl of FBS per flask and were incubated for 20 minutes. After incubation, 5 ml of supplemented DMEM as already described was added to each culture, and the culture conditions were maintained as described for the SF samples.

### Trypsinization and doubling time

Cells in all cultures were grown in monolayers under standard sterile conditions until reaching >80 % confluence and were then trypsinized. DMEM was removed, and the cells were washed with 2 ml of PBS. Thereafter, 1 ml of 0.25 % trypsin was added to each flask, and the samples were incubated at 37 °C for 5 minutes. Subsequently, 2 ml of culture medium supplemented with FBS was added to inactivate the trypsin. The cell suspension was then aspirated and transferred into a 15 ml conical tube for centrifugation at 287 × *g* for 5 minutes to remove the trypsin. For each sample, the cell pellet was resuspended in 1 ml of supplemented DMEM, and an aliquot of 10 μl was used for cell counting in a Neubauer chamber. The remaining cells were transferred into a 75 cm^2^ flask to which 9 ml of medium was added, and cells were incubated under the conditions already described (considered first passage (P1)). Calculation of the doubling time (DT) of the mesenchymal cells from SF-H, SF-OCD, and SF-OA was performed using an algorithm available online [24], accounting for cell number at P1, second passage (P2), and third passage (P3) during the exponential growth phase. The formula used by the online tool was:

$$ \mathrm{D}\mathrm{T} = t\kern0.5em  \times \log 2\ /\ \left( \log N\mathrm{t}\kern0.5em /\  \log N0\right) $$

where *N*0 is the initial number of cells plated, *Nt* is the number of cells at the end of the incubation time, and *t* is the incubation time in hours. For SMs (SM-H, SM-OCD, and SM-OA), only the size of the fragment (in milligrams) was known, rather than the initial numbers of cells, so the initial cell numbers were estimated based on the days required for passages (>80 % confluence).

### Immunophenotyping characterization

#### Flow cytometry

Using a FACSCalibur® cytometer (Becton Dickinson, San Jose, CA, USA) and Cell-Quest software (Becton Dickinson, San Jose, CA, USA), phenotypic assessment of SF-H (*n* = 14), SF-OCD (*n* = 21), SF-OA (*n* = 16), SM-H (*n* = 16), SM-OCD (*n* = 16), and SM-OA (*n* = 11) was performed analyzing 5000 cells per group at P3 for all joint conditions. Mouse anti-rat CD90-phycoerythrin (PE) (clone OX-7; BD, San Jose, CA, USA), mouse anti-horse CD44-fluorescein isothiocyanate (FITC) (clone CVS18; AbD Serotec, Oxford, UK), mouse anti-human CD105-RPE (clone SN6; AbD Serotec, Oxford, UK), and mouse anti-human CD34-FITC (clone 581; BD) antibodies were used. Anti-IgG1-PE and anti-IgG1-FITC were used as control isotypes to calibrate the cytometer. The protocols were performed according to the manufacturer’s instructions.

#### Immunocytochemistry

At P3, samples were plated in six-well plates (TPP; Trasadingen, Switzerland), and 3 ml of supplemented DMEM was added per well. After the cells reached ≥80 % confluence, the DMEM was removed, and the plates were washed twice with 2 ml of PBS. Next, the cells were fixed in 4 % paraformaldehyde at 4 °C for 30 minutes. After fixation, the plates were washed again and then incubated with the following primary antibodies: rabbit anti-human lysozyme (Dako; Carpinteria, California, USA), rabbit anti-human PGP 9.5 (Spring Bioscience; Pleasanton, California, USA), rabbit anti-human Oct4 (Biorbyt; Berkeley, California, USA), goat anti-human nanog (clone N-17; Santa Cruz Biotechnology; Santa Cruz, California, USA), mouse anti-human vimentin (clone V9; Dako; Carpinteria, California, USA), mouse anti-human cytokeratin (clones EA-1 and AE3; Dako; Carpinteria, California, USA), and mouse anti-human proliferating cell nuclear antigen (PCNA) (clone PC10; Dako; Carpinteria, California, USA ). The plates were incubated at 4 °C overnight. Super-Picture polymer was used to detect the primary antibodies, and the reactions were revealed using diaminobenzidine solution (DAB, Sigma, St. Louis, MO, USA) and counterstained with Harris hematoxylin.

#### Chondrogenic, osteogenic, and adipogenic cell differentiation (SF and SM)

The chondrogenic induction was prepared from a solution containing 2 × 10^6^ cells of pellet cultures during P3, and cells were cultured in a conical tube with 2 ml of DMEM for 48 hours. The inductive phase was initiated after the medium was changed from maintenance medium to a commercial chondrogenic inducer medium (StemPro chondrogenesis kit; GIBCO, Carlsbad, California, USA). The differentiation medium was changed every 48 hours for a course of 21 days. After this period, the pellets were washed with PBS and fixed in 4 % paraformaldehyde for 24 hours. To confirm chondrogenic differentiation, histological slides were prepared, and pellets were stained with toluidine blue, alcian blue, and hematoxylin and eosin (H & E; Sigma-Aldrich Corp., St. Louis, MO, USA).

For osteogenic and adipogenic differentiation, cells at P3 were placed in plastic six-well plates at a concentration of 10^5^ cells per well. After the cells had adhered to the plastic, 2 ml of supplemented DMEM was added per well for a period of 48 hours. Next, the DMEM was replaced with either the commercial osteogenic inducer medium (StemPro osteogenesis kit; GIBCO) or the commercial adipogenic inducer medium (StemPro adipogenesis kit; GIBCO), and the medium was changed every 48 hours for 21 or 14 days, respectively. After the differentiation period, the plates were washed twice with PBS. Osteogenic differentiation was confirmed by positive staining of the extracellular calcium matrix using 2 % Alizarin Red at pH 4.2 (Sigma-Aldrich Corp.). Adipogenic differentiation was confirmed by the deposition of lipid droplets in the cytoplasm using Oil Red O (Sigma-Aldrich Corp.) and staining of the cell nuclei using H & E. The analysis of control cells, which received no inducing medium, was conducted following their culture in DMEM under the same timing and staining conditions as already described.

Quantification of positive matrix area was performed using AxioVision LE64 software (Carl Zeiss, Oberkochen, Germany). The program analyzed 10 photographs of each plate (magnification = 10×) and calculated the area of positive matrix in square micrometers (μm^2^) for osteogenic differentiation and square centimeters (cm^2^) for chondrogenic differentiation. The intensity of adipogenic differentiation was assessed using a scoring system based on Oil Red O staining (Table 1) [25].

**Table 1:** Semiquantitative scoring system used in the evaluation of adipogenic differentiation

Score	% of differentiated cells	Size and arrangement of lipid droplets
0	0–5	No droplets
1	>5–50	Isolated and small
2	>50–80	Medium sized
3	>80–100	Predominantly large

#### Tumorigenicity test

A tumorigenicity test was performed using nine immunosuppressed Balb-C^nu/nu^ female mice, each approximately 6 months old and weighing 19–28 g.

Cells that were cultured from SF and SM taken from healthy and diseased joints (OCD and OA) were grown in culture to P3 and then injected into dorsal subcutaneous tissue in mice at a density of 10^6^ cells per animal. After 3 months, the mice were sacrificed by intraperitoneal administration of xylazine, followed by a 10-minute-long exposure to CO_2_. Necropsy was performed, and lung, kidney, liver, subcutaneous tissue, and spleen samples were collected, weighed, fixed in 10 % formalin, and sent for histological analysis. The samples were processed by a paraffin inclusion technique and were stained with H & E.

### Statistical analysis

The data were analyzed using GraphPad Prism 6 (GraphPad Software Inc., San Diego, CA, USA). Significant differences between groups were determined using one-way analysis of variance (ANOVA) followed by Dunnett’s test. All data are expressed as the mean ± standard deviation (SD), and the level of significance was set at *P* <0.05.

## Results

### Cell culture and doubling time

**Fig. 1:**
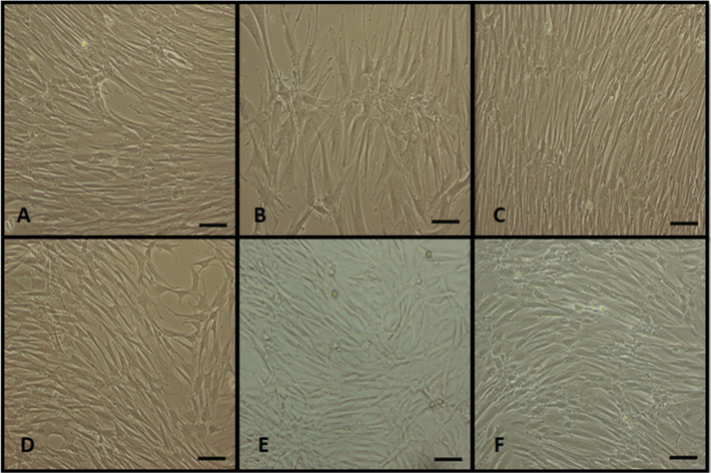
MSCs from synovial tissues during cell culture (P3) showing ≥80 % confluence. SF-H **a**, SF-OCD **b**, SF-OA **c**, SM-H **d**, SM-OCD **e**, and SM-OA **f**. 100× magnification

MSCs that were cultured from SF exhibited the capacity to adhere to plastic after 4–7 days in culture. Meanwhile, MSCs that were derived from SM adhered to the flasks after 15 days of culture. Both populations had monolayer growth profiles, morphologically resembled fibroblasts (Fig. 1), and maintained this appearance after long-term culture (data not shown).

**Fig. 2:**
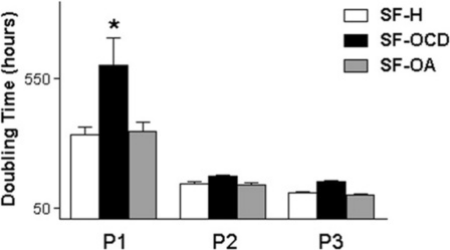
Graph showing the DT (mean ± SD) from SFs (SF-H, SF-OCD, and SF-OA) during P1, P2, and P3. *P <0.05. *P1* first passage, *P2* second passage, *P3* third passage, *SF-H* synovial fluid from healthy joints, *SF-OA* synovial fluid from joints with osteoarthritis, *SF-OCD* synovial fluid from joints with osteochondritis dissecans

The doubling times for SF-H, SF-OCD, and SF-OA were, respectively, 334 ± 64, 585 ± 73, and 333 ± 70 hours at P1; 144 ± 24, 162 ± 23, and 134 ± 20 hours at P2; and 108 ± 12, 144 ± 13, and 98 ± 8 hours at P3. At P1, one-way ANOVA revealed a significant difference in doubling time, and the Tukey–Kramer test indicated a significant increase in the doubling time of SF-OCD compared with the SF-H and SF-OA (*P* <0.05). However, there were no evident differences at P2 or P3 (Fig. 2).

The timing to reach 80 % confluence during primary culture varied among the SM samples: 45 days for SM-H, 38 days for SM-OCD, and 35 days for SM-OA. The doubling time of SF and the days for passage of SM could not be compared because the methods for analysis differed between these conditions. After P1, following the trypsinization protocol, 80 % confluence was achieved at an average of 11 days for both groups (SF and SM).

The time that elapsed between primary culture and P3, when phenotypic characterization and cell differentiation were performed, was approximately 73 days for SF-H, 89 days for SF-OCD, 60 days for SF-OA, 68 days for SM-H, 57 days for SM-OCD, and 54 days for SM-OA.

### Phenotypic characterization

#### Flow cytometry

Flow cytometric analysis at P3 indicated that the cells that were cultured from SF and SM exhibited phenotypic characteristics that were consistent with those of MSCs, and there were no cells of hematopoietic origin because the cells exhibited positive expression of the markers CD90 and CD44 and no expression of the markers CD105 and CD34.

**Fig. 3:**
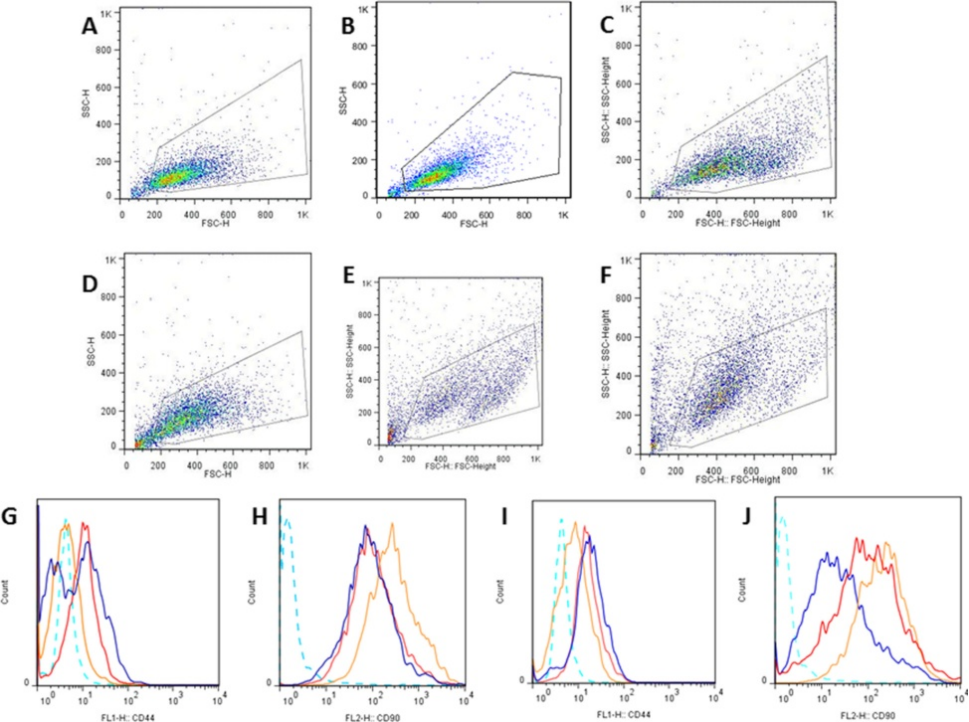
Flow cytometric analysis of the expression of cell surface markers CD90 and CD44 by MSCs. Representative dot plots and histograms (overlaid) of six different synovial sources: SF-H **a**, SF-OCD **b**, SF-OA **c**, SM-H **d**, SM-OCD **e**, and SM-OA **f** analyzed during the third passage; overlaid CD44^+^ cells from SF-H, SF-OCD, and SF-OA **g**; overlaid CD90^+^ cells from SF-H, SF-OCD, and SF-OA **h**; overlaid CD44^+^ cells from SM-H, SM-OCD, and SM-OA **i**; and overlaid CD90^+^ cells from SM-H, SM-OCD, and SM-OA **j** (*orange line*, SF-H and SM-H; *red line*, SF-OCD and SM-OCD; *blue line*, SF-OA and SM-OA). Isotype control antibodies were used (*blue dotted line*). All sources showed significant expression of mesenchymal markers (CD90 and CD44). (Color figure online). SSC: side scatter; FSC: forward scatter

Table 2 presents the average (SD) percentages for each of the markers from the different sources of MSCs. Significantly higher proportions of double-positive cells (CD44^+^CD90^+^) were observed for SF-OCD (16.5 ± 16.8) and SF-OA (11.2 ± 10.8) than for SF-H (6.65 ± 8.86) (*P* <0.05). For SM groups, the same increase was observed but for SM-OA (11.2 ± 10) compared with SM-OCD (8.9 ± 18.5) and SM-H (2.2 ± 2.96). Figure 3 shows a dot plot of the population chosen (gated cell population) and overlaid histogram analysis representatives from different synovial source.

**Table 2:** Average percentages of MSCs from SF and SM that exhibited positive or negative expression of CD90, CD44, CD105, and CD34 markers by flow cytometry

Source	Expression			
	CD90^+^CD44^–^	CD90^–^CD44^+^	CD90^–^CD44^–^	CD44^+^CD90^+^
SF-H	64.9 ± 23.8^a^	1.18 ± 1.4^a^	27.3 ± 22.3	6.65 ± 8.86^a^
SF-OCD	48.3 ± 26.3^b^	3.98 ± 6^b^	31.2 ± 24.7	16.5 ± 16.8^b^
SF-OA	48.1 ± 23^b^	14.2 ± 25.7^c^	26.5 ± 19.2	11.2 ± 10.8^b^
SM-H	66.6 ± 30.1^a^	1.49 ± 3.2^a^	29.7 ± 27.6	2.2 ± 2.96^a^
SM-OCD	40.2 ± 27.2^b^	2.17 ± 2.6^a^	48.7 ± 30.1	8.9 ± 18.5^a,b^
SM-OA	40.3 ± 22.1^b^	8.56 ± 9.2^b^	39.9 ± 15.4	11.2 ± 10^b^
	CD105^+^CD34^–^	CD105^–^CD34^+^	CD105^–^CD34^–^	CD105^+^CD34^+^
SF-H	0.25 ± 0.49	0.02 ± 0.04	99.7 ± 0.50	0.03 ± 0.04
SF-OCD	0.22 ± 0.48	0.09 ± 0.29	98.7 ± 3.07	0.96 ± 2.85
SF-OA	0.25 ± 0.50	0.11 ± 0.37	99.3 ± 1.36	0.37 ± 0.82
SM-H	0.28 ± 0.76	0.02 ± 0.03	99.7 ± 0.79	0.02 ± 0.03
SM-OCD	0.36 ± 0.83	0.10 ± 0.27	99.4 ± 1.02	0.17 ± 0.28
SM-OA	0.36 ± 0.54	0.04 ± 0.12	99.4 ± 0.56	0.11 ± 0.14

Data presented as mean ± standard deviation. Different superscript letters in the same column denote statistically significant differences (*P* <0.05)

*SF-H* healthy synovial fluid, *SF-OA* osteoarthritis synovial fluid, *SF-OCD* osteochondritis dissecans synovial fluid, *SM-H* healthy synovial membrane, *SM-OA* osteoarthritis synovial membrane, *SM-OCD* osteochondritis dissecans synovial membrane

#### Immunocytochemistry

**Fig. 4:**
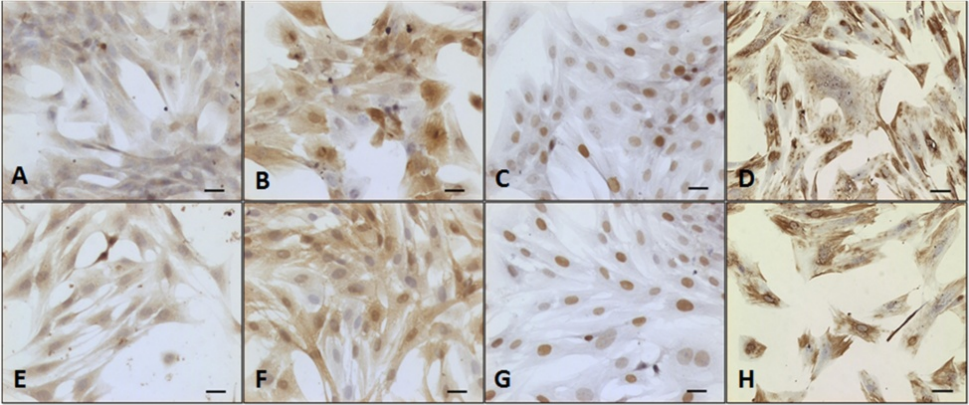
Representative images of lysozyme, PGP 9.5, PCNA, and vimentin expression in synovial tissues using immunocytochemistry. Immunocytochemistry of cells from equine SF **a**–**d** and SM **e**–**h** during their third passage to evaluate the positive expression of the cell surface markers lysozyme **a**, **e**, PGP 9.5 **b**, **f**, PCNA **c**, **g**, and vimentin **d**, **h**. 200× magnification

Immunocytochemistry analysis confirmed positive immunostaining for lysozyme, PGP 9.5, PCNA, and vimentin in the cells from the SF and SM groups, which ensured the existence of type A and B synoviocytes, intense cell proliferation, and the presence of MSCs, respectively (Fig. 4). The absence of pluripotent cells and fibroblasts was confirmed by negative immunostaining for Oct4, cytokeratin, and nanog.

#### Differentiation potential

**Fig. 5:**
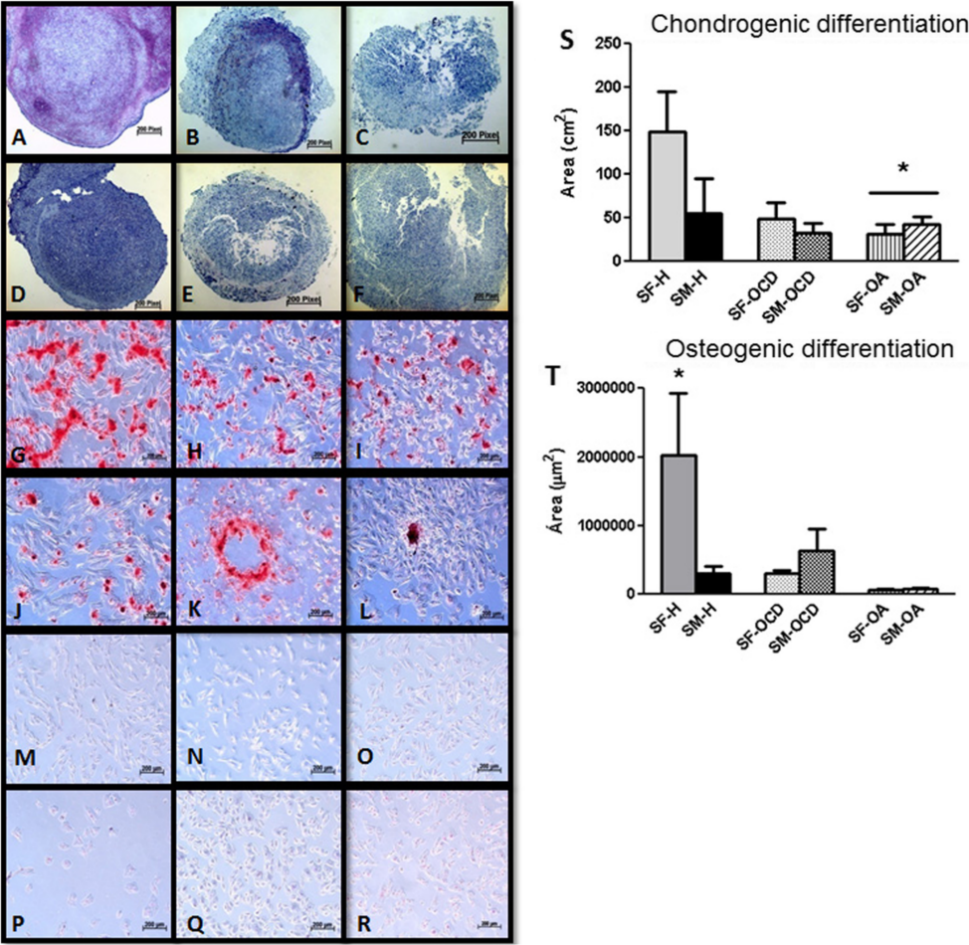
Differentiation potential of MSCs from equine SF-H, SF-OCD, SF-OA, SM-H, SM-OCD, and SM-OA. Differentiation of MSCs from SF and SM into mesenchymal lineages during P3. MSCs after chondrogenic differentiation stained with toluidine blue showing hyaline matrix (*blue*): SF-H **a**, SF-OCD **b**, SF-OA **c**, SM-H **d**, SM-OCD **e**, and SM-OA **f** (400× magnification). MSCs stained with Alizarin Red showing matrix calcium formation, confirming the osteogenic lineage (*red*): SF-H **g**, SF-OCD **h**, SF-OA **i**, SM-H **j**, SM-OCD **k**, and SM-OA **l**. MSCs showing intracytoplasmic lipid droplets confirming the adipogenic lineage: SF-H **m**, SF-OCD **n**, SF-OA **o**, SM-H **p**, SM-OCD **q**, and SM-OA **r** (1000×). Chondrogenic differentiation area **s**. Osteogenic differentiation area **t**. Quantification of positive matrix area was performed using AxioVision LE64 software (Carl Zeiss). The program analyzed 10 photographs of each plate (magnification = 10×) and calculated the area of positive matrix when blue and red stains for chondrogenic and osteogenic differentiation, respectively, were observed. *P ˂0.05. *SF-H* synovial fluid from healthy joints, *SF-OA* synovial fluid from joints with osteoarthritis, *SF-OCD* synovial fluid from joints with osteochondritis dissecans, *SM-H* synovial membrane from healthy joints, *SM-OA* synovial membrane from joints with osteoarthritis, *SM-OCD* synovial membrane from joints with osteochondritis dissecans (Color figure online)AU Query: Confirm Fig 5 caption after editing to style OK

Chondrogenic differentiation was observed after 21 days in MSCs from both of the groups (SF and SM), at which point the formation of spherical pellets of hardened appearance was observed. Chondrogenic potential was confirmed by alcian blue, toluidine blue (Fig. 5), and H & E staining, which enabled the identification of an extracellular matrix rich in proteoglycans and of cells such as chondrocytes by optical microscopy.

Differences in staining were noticeable in both groups (SF and SM). Slides containing healthy SF material exhibited much more evident staining and better morphology than slides containing OCD material, which in turn exhibited more evident staining than slides containing OA material.

The SF-H (149 ± 103 cm^2^) and SM-H (78 ± 7 cm^2^) groups showed larger average areas of positive staining for extracellular matrix than did the SF-OA (32 ± 22 cm^2^) and SM-OA (43 ± 20 cm^2^) groups (*P* <0.05), respectively, but the SF-OCD (49 ± 41 cm^2^) and SM-OCD (32 ± 20 cm^2^) groups appeared similar to the SF-H and SM-H groups (Fig. 5).

In both groups (SF and SM), osteogenic differentiation occurred after 21 days of induction. Osteogenic differentiation potential was confirmed by positive staining of the calcium matrix by Alizarin Red (Fig. 5). The cells in control culture did not form a calcium matrix, as certified by negative staining with Alizarin Red.

The average and standard deviation of mineralized matrix areas for SF-H (1,105,447 ± 1,415,829 μm^2^) were larger than for SF-OA (83,765 ± 48,589 μm^2^), SF-OCD (295,566 ± 120,472 μm^2^), SM-H (166,783 ± 193,938 μm^2^), SM-OA (141,648 ± 123,734 μm^2^), and SM-OCD (265,098 ± 174,578 μm^2^) (Fig. 5).

Few cell colonies underwent adipogenic differentiation by 14 days after induction. This ability appeared to be limited for this lineage, and the results were similar among the groups. Adipogenic differentiation was visualized at small isolated points at the edges of the plates, but cell death was observed after the induction of differentiation (i.e., the cells detached from the plates). Adipogenic differentiation potential was confirmed after observation of a morphology change from fusiform to polygon and by the deposition of lipid droplets in the cytoplasm, which were stained by Oil Red O (Fig. 5), and each group reached a score of 1 (showing <20 % positive cells). The control population did not undergo the morphological change and exhibited negative staining.

#### Tumorigenic potential

None of the mice that received subcutaneous injections of MSCs from synovial tissues exhibited any changes in behavior, appetite, body temperature, or local inoculation temperature throughout the experimental period.

**Fig. 6:**
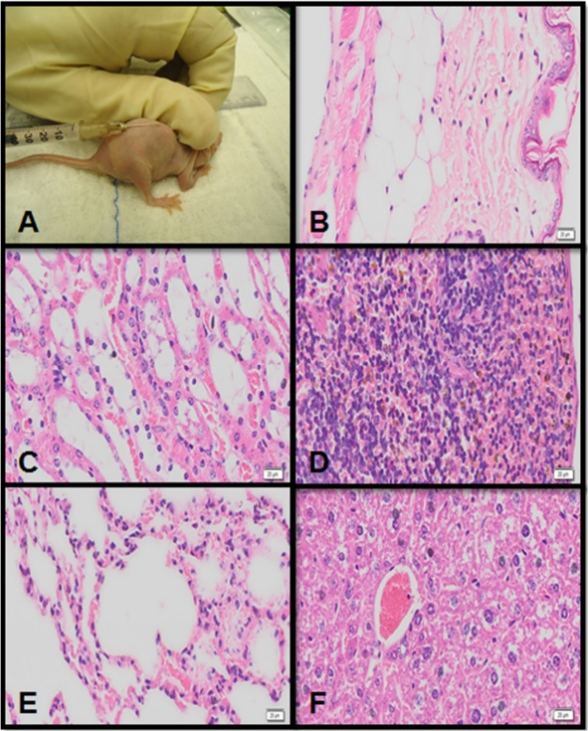
Histologic evaluation of tumorigenicity test after subcutaneous injection of P3 cells into Balb-C^nu/nu^ mice. MSC inoculation **a**, and subcutaneous tissue **b**, kidney **c**, spleen **d**, lung **e** and liver **f** tissue. The results of tumorigenicity tests in mice showed no compromise of any internal organ, assuring the applicability of the studied cells. 1000× magnification

A necropsy evaluation revealed no macroscopic changes; organs and tissue samples were collected (liver, lung, kidney, spleen, and subcutaneous tissue) and were sent for histological analysis. No changes in tissue characteristics or cell morphology were observed (Fig. 6). These results demonstrated that MSCs from synovial tissues are unable to induce tumor formation and indicate the safety of their clinical applicability.

## Discussion

In this study, cells from synovial tissues were evaluated as possible sources of MSCs, and their suitability was assessed based on their expression of surface markers and their ability to differentiate into various mesenchymal lineages. Subsequently, cells that were cultured from healthy joints, joints with OA, and joints with OCD were compared to identify the best candidate for future clinical applications.

De Bari et al. [4] were the first to isolate MSCs from synovial tissues. They harvested SM from human patients with degenerative joint disease and used collagenase to extract cells from tissues. Our study was similar with respect to the aseptic harvesting of cells from diseased (OCD and OA) and healthy joints during surgery. We observed cell growth in monolayers in all of the samples from SM, although enzymatic digestion with collagenase was not performed.

In our experiment, the first passage of MSCs from SM took approximately 15 days, and another 45 days of incubation time was require for the cells to reach 80 % confluence. This delay could be associated with the fact that collagenase was not used—as collagenase has been used in all related studies, in which cell expansion and cell confluence were observed by the second week [26, 27].

All of the MSCs evaluated in this study required a long period of time from primary culture until P3. Typically, equine MSCs are derived from other sources, such as bone marrow and adipose tissue, and require 15–25 days to reach P3 [4]. A relevant result observed was an increased time to reach confluence for the SF-OCD group at P1 in relation to other synovial sources, but this increased time was not observed for later passages.

All of the cultures from synovial tissues exhibited plastic adherence capacity, monolayer growth, and fibroblastoid morphology. The processing was easier for SF cells than for SM cells. These results are consistent with findings from other studies [17, 26, 28, 29].

The criteria for characterization of MSCs from horses are based on a marker panel [30] and include several of the criteria that are used to characterize human MSCs, as determined by the International Society for Cellular Therapy [31]. The current study examined cells for the expression of the surface markers CD90, CD44, CD34, CD105, Oct4, nanog, vimentin, and cytokeratin to establish phenotypic expression patterns of SF and SM cells. Furthermore, the choice of the markers was based on previous studies on equine MSCs from other sources that used several of these markers [4, 7, 32, 33].

MSCs from both SF and SM sources exhibited positive expression for the marker CD90. Higher proportions of cells showing positive expression for CD90 were found in SF-H (64.9 %) and SM-H (66.6 %) (*P* <0.05), suggesting that cells from healthy SF or SM are better sources of MSCs than are cells from SF-OCD (48.3 %), SF-OA (48.1 %), SM-OCD (40.2 %), or SM-OA (40.3 %).

A surprisingly low expression rate of the marker CD44 was observed. Similar results have been observed by Ranera et al. [7], who demonstrated an absence of CD44 expression on cells from bone marrow and adipose tissue. In the present study, it is important to note that double staining was performed and that competition for epitopes could have occurred, potentially showing greater expression of CD90 and lower expression of CD44. Nevertheless, all of the cell populations exhibited positive expression of CD44, which was more strongly expressed in the OCD and OA groups than in the groups from healthy joints (*P* <0.05).

Interestingly, cells derived from OA exhibited greater proliferation potential and higher proportions of CD44^+^CD90^+^ double-positive cell expression than cells derived from healthy tissue. According to Kobayashi et al. [34], the identification of multipotent CD44^+^/CD90^+^ stem cells with high proliferative potential suggested that these cells could provide a basis for continuously self-renewing cartilage.

Many markers have been frequently tested for use in horses because of a lack of specific equine antibodies and the low reactivity levels of markers from other species against equine proteins [30]. Therefore, the lack of CD105 expression observed in the present study may be related to a low specificity to equine CD105. Additionally, De Schauwer et al. [30] observed large variations in CD105 expression (0.1–20 %) among umbilical cord blood samples, and another study on umbilical cord tissue reported negative expression of CD105 [35], possibly because the antibody that was used did not work for the cells that were studied.

In the present study, flow cytometry analysis of the marker CD34 was performed to investigate the presence of hematopoietic cells in SF and SM cultures. Expression of CD34 was not found in any of the analyses (0.02 % SF-H, 0.09 % SF-OCD, 0.11 % SF-OA, 0.02 % SM-H, 0.10 % SM-OCD, and 0.04 % SM-OA). These results are similar to findings from several previously conducted studies on human cells in which MSCs from SF and SM have exhibited either low or no expression of CD34 [36–39]. Only one study has been published that used equine MSCs from SF, and it indicated that there was no expression of CD34 in these cells [12].

Kitamura et al. [40] reported positive immunostaining of the marker PGP 9.5 in type B synoviocytes, clearly documenting their distribution in SM. This specificity in identifying type B synoviocytes was allowed by the comparison of immunoreactions in nerve fibers that are distributed throughout the SM with results obtained from human brain and horse samples. Type A synoviocytes are similar to macrophages and are present in SF [41].

The immunocytochemistry data showed conservation of type A and B synoviocytes in cell cultures from SF and SM from healthy and diseased joints (OCD and OA) by positive immunostaining for lysozyme and PGP 9.5. Type A and B synoviocytes were also observed by Sakaguchi et al. [36] during culture of cells isolated from synovium; their data suggested that nucleated cells after isolation or digestion may lose their original profiles and acquire MSC profiles during the expansion of the MSCs.

Immunocytochemistry analysis mainly showed positive expression of vimentin and PCNA and an absence of expression of cytokeratin. These results suggest mesenchymal origin and proliferative abilities for all of the samples.

Additionally, there was an absence of Oct4 expression in our study. These results were similar to those found in a study by De Vita et al. [42] that evaluated horse amniotic fluid; however, they conflicted with results reported in a study that employed MSCs from goat amniotic fluid [43] and results from a study that employed MSCs from horse bone marrow, umbilical cord matrix, and amniotic fluid [34]. Our results suggest that the studied cells lack pluripotency. MSCs from intervascular and perivascular umbilical cord matrix in horses showed positive expression of Oct4 [35], as did MSCs from human bone marrow and amniotic membrane [10]. Gao et al. [44] demonstrated positive expression of Oct4 and nanog in MSCs from human adipose tissue by immunocytochemistry and reverse transcription PCR.

MSCs are characterized by an extensive proliferative ability, as well as by the ability to differentiate in vitro into various mesenchymal lineages in response to appropriate stimuli [4]. In this study, both types of synovial tissues (SF and SM) were able to differentiate into osteoblasts and chondrocytes.

Slides containing healthy SF material exhibited much more evident staining and better morphology than slides containing OCD and OA materials, but the average areas showing positive staining for extracellular matrix in the SF-H and SM-H groups were only higher than those of the SF-OA and SM-OA groups. These data were consistent with the flow cytometric findings showing higher positive expression of CD90 in SF and SM from healthy joints compared with tissues from OCD or OA joints. These results suggest that healthy equine SF and SM represent an attractive source of MSCs for therapeutic use in osteoarticular diseases.

Currently, several sources have been reported for obtaining MSCs; however, few studies have compared synovial tissue with other type of sources. Yoshimura et al. [45] compared the performance, proliferation capacity, and chondrogenic differentiation potential of MSCs derived from bone marrow, SM, periosteum, fat, and muscle. These authors demonstrated a higher chondrogenic potential in MSCs from SM due to the increased production of cartilage matrix in relation to other sources. Similarly, Mochizuki et al. [14] compared MSCs from SM and adipose tissue (self-renewal and differentiation capacity), which also showed a better chondrogenic capacity of MSCs derived from SM. It was inferred that these cells are already predisposed to differentiate into chondrocytes, suggesting that the ancestral microenvironment directs the “destination” cell upon differentiation.

The cells cultured from SF and SM showed little ability in adipogenic differentiation, as demonstrated by their limited capacity to produce intracytoplasmic lipid droplets, in addition to undergoing cell death during the process of induction. Nevertheless, there were changes in cell morphology which suggested that they have the capacity for differentiation into adipocytes.

Regardless of the source of MSCs, the process of adipogenic differentiation in equine cells appears to be dependent on components found in rabbit serum [25]. Koch et al. [9] and Giovannini et al. [46] found that adipogenic differentiation in equine MSCs which were derived from umbilical cord blood or peripheral blood was limited when they were induced in standard culture medium but that the differentiation occurred when the medium was supplemented with rabbit serum. These observations may explain the low differentiation rate found in the current study, as the adipogenic differentiation was induced using a standard commercial environment that was not supplemented with rabbit serum.

Tumorigenic analysis showed that MSCs from equine SF and SM were not able to induce tumors in nude mice, which was confirmed during necropsy and in histological slides. This is the first study to demonstrate that MSCs from equine SF and SM are safe, as they were unable to cause tumor growth in a laboratory animal model. Previous studies that have shown the safety and immunomodulatory characteristics of MSCs from other sources were based on experiments that were conducted in vitro and that focused on the suppression of B-lymphocyte activity [47–50]. The negative results obtained in the tumorigenicity test conducted in this study further encourage safety of studies in equines.

Regarding the clinical routine, SF is easier to obtain than SM; furthermore, SF exhibited negative tumorigenicity and favorable results during phenotypic characterization and cell differentiation. Based on these results, we consider that the clinical applicability of allogeneic MSCs from healthy SF must be tested in healthy horses. However, other authors have observed that allogeneic MSCs are capable of eliciting antibody responses in vivo (intradermal injection). It has been suggested that such responses could limit the effectiveness of repeated use of allogeneic MSCs in a single horse and could also result in harmful inflammatory responses in recipients [51]. Further studies are necessary to analyze whether allogeneic MSCs injected into equine joint can also be immunogenic.

## Conclusions

SF and SM from healthy or diseased joints (OA and OCD) are feasible sources for harvesting equine MSCs based on results confirming the phenotypic and multipotentiality characteristics of these cells. All sources studied provide suitable MSCs for an allogeneic therapy cell bank; nevertheless, MSCs from healthy joints may be preferable for cell banking purposes because they exhibit better chondrogenic differentiation capacity than MSCs derived from diseased joints.

The tumorigenicity test showed that MSCs from SF and SM can be used in clinical trials because they lack the potential to form teratomas.

## Abbreviations

ANOVA: Analysis of variance
DMEM: Dulbecco’s modified Eagle medium
DT: Doubling time
FBS: Fetal bovine serum
FITC: Fluorescein isothiocyanate
H % E: Hematoxylin and eosin
MSC: Mesenchymal stem cell
OA: Osteoarthritis
OCD: Osteochondritis dissecans
P1: First passage
P2: Second passage
P3: Third passage
PBS: Phosphate-buffered saline
PCNA: Proliferating cell nuclear antigen
PE: Phycoerythrin
SD: Standard deviation
SF: Synovial fluid
SF-H: Synovial fluid from healthy joints
SF-MSC: Mesenchymal stem cell from synovial fluid
SF-OA: Synovial fluid from joints with osteoarthritis
SF-OCD: Synovial fluid from joints with osteochondritis dissecans
SM: Synovial membrane
SM-H: Synovial membrane from healthy joints
SM-MSC: Mesenchymal stem cell from synovial membrane
SM-OA: Synovial membrane from joints with osteoarthritis
SM-OCD: Synovial membrane from joints with osteochondritis dissecans

## References

[1] Pittenger MF, Mackay AM, Beck SC, Jaiswal RK, Douglas R, Mosca JD (1999). Multilineage potential of adult human mesenchymal stem cells. Science.

[2] Rozemuller H, Prins HJ, Naaijkens B, Staal J, Bühring HJ, Martens AC (2010). Prospective isolation of mesenchymal stem cells from multiple mammalian species using cross-reacting anti-human monoclonal antibodies. Stem Cells Dev.

[3] Barberini DJ, Freitas NPP, Magnoni MS, Maia L, Listoni AJ, Heckler MC (2014). Equine mesenchymal stem cells from bone marrow, adipose tissue and umbilical cord: immunophenotypic characterization and differentiation potential. Stem Cell Res Ther.

[4] De Bari C, Dell’Accio F, Tylzanowski P, Luyten FP (2001). Multipotent mesenchymal stem cells from adult human synovial membrane. Arthritis Rheum.

[5] De Mattos AC, Alves ALG, Golim MA, Moroz A, Hussni CA, De Oliveira PGG (2009). Isolation and immunophenotypic characterization of mesenchymal stem cells derived from equine species adipose tissue. Vet Immunol Immunopathol.

[6] Toupadakis CA, Wong A, Genetos DC, Cheung WK, Borjesson DL, Ferraro GL (2010). Comparison of the osteogenic potential of equine mesenchymal stem cells from bone marrow, adipose tissue, umbilical cord blood, and umbilical cord tissue. Am J Vet Res.

[7] Ranera B, Lyahyai J, Romero A, Vázquez FJ, Remacha AR, Bernal ML (2011). Immunophenotype and gene expression profiles of cell surface markers of mesenchymal stem cells derived from equine bone marrow and adipose tissue. Vet Immunol Immunopathol.

[8] Carrade DD, Lame MW, Kent MS, Clark KC, Walker NJ, Borjesson DL (2012). Comparative analysis of the immunomodulatory properties of equine adult-derived mesenchymal stem cells. Cell Med.

[9] Koch TG, Heerkens T, Thomsen PD, Betts DH (2007). Isolation of mesenchymal stem cells from equine umbilical cord blood. BMC Biotechnol.

[10] Lange-Consiglio A, Corradetti B, Meucci A, Perego R, Bizzaro D, Cremonesi F (2013). Characteristics of equine mesenchymal stem cells derived from amnion and bone marrow: in vitro proliferative and multilineage potential assessment. Equine Vet J.

[11] Koerner J, Nesic D, Romero JD, Brehn W, Mainil-Varlet P, Grogan SP (2006). Equine peripheral blood-derived progenitors in comparison to bone marrow-derived mesenchymal stem cells. Stem Cells.

[12] Murata D, Miyakoshi D, Hatazoe T, Miura N, Tokunaga S, Fujiki M (2014). Multipotency of equine mesenchymal stem cells derived from synovial fluid. Vet J.

[13] Prado AAF, Favaron PO, Silva LCLC, Baccarin RYA, Miglino MA, Maria DA (2015). Characterization of mesenchymal stem cells derived from the equine synovial fluid and membrane. BMC Vet Res.

[14] Mochizuki T, Muneta T, Sakaguchi Y, Nimura A, Yokoyama A, Koga H (2006). Higher chondrogenic potential of fibrous synovium- and adipose synovium-derived cells compared with subcutaneous fat-derived cells: Distinguishing properties of mesenchymal stem cells in humans. Arthritis Rheum.

[15] Arufe MC, De La Fuente A, Fuentes-Boquete I, De Toro FJ, Blanco FJ (2009). Differentiation of synovial CD-105+ human mesenchymal stem cells into chondrocyte-like cells through spheroid formation. J Cell Biochem.

[16] Jones E (2011). Synovial mesenchymal stem cells in vivo: potential key players for joint regeneration. World J Rheumatol.

[17] Krawetz RJ, Wu YE, Martin L, Rattner JB, Matyas JR, Hart DA (2012). Synovial fluid progenitors expressing CD90+ from normal but not osteoarthritic joints undergo chondrogenic differentiation without micro-mass culture. PLoS One.

[18] McGonagle D, Jones E (2008). A potential role for synovial fluid mesenchymal stem cells in ligament regeneration. Rheumatology.

[19] Hermida-Gómez T, Fuentes-Boquete I, Gimeno-Longas MJ, Muiños-López E, Díaz-Prado S, De Toro FJ (2011). Quantification of cells expressing mesenchymal stem cell markers in healthy and osteoarthritic synovial membranes. J Rheumatol.

[20] Lee DH, Sonn CH, Han SB, Oh Y, Lee KM, Lee SH (2012). Synovial fluid CD34–CD44 + CD90+ mesenchymal stem cell levels are associated with the severity of primary knee osteoarthritis. Osteoarthritis Cartilage.

[21] Lee JC, Lee SY, Min HJ, Han SA, Jang J, Lee S (2012). Synovium-derived mesenchymal stem cells encapsulated in a novel injectable gel can repair osteochondral defects in a rabbit model. Tissue Eng Part A.

[22] Santhagunam A, Santos FD, Madeira C, Salgueiro JB, Cabral JMS (2014). Isolation and ex vivo expansion of synovial mesenchymal stromal cells for cartilage repair. Cytotherapy.

[23] Morito T, Muneta T, Hara K, Ju YJ, Mochizuki T, Makino H (2008). Synovial fluid-derived mesenchymal stem cells increase after intra-articular ligament injury in humans. Rheumatology.

[24] Roth V. 2006. http://www.doubling-time.com/compute.php. Accessed 3 Nov 2015.

[25] Burk J, Ribitsch I, Gittel C, Juelke H, Kasper C, Staszyk C, Brehm W (2013). Growth and differentiation characteristics of equine mesenchymal stroma cells derived from different sources. Vet J.

[26] Koga H, Muneta T, Ju YJ, Nagase T, Nimura A, Mochizuki T (2007). Synovial stem cells are regionally specified according to local microenvironments after implantation for cartilage regeneration. Stem Cells.

[27] Kurth T, Hedbom E, Shintani N, Sugimoto M, Chen FH, Haspl M (2007). Chondrogenic potential of human synovial mesenchymal stem cells in alginate. Osteoarthritis Cartilage.

[28] Jones EA, English A, Henshaw K, Kinsey SE, Markham AF, Emery P (2004). Enumeration and phenotypic characterization of synovial fluid multipotential mesenchymal progenitor cells in inflammatory and degenerative arthritis. Arthritis Rheum.

[29] Matsukura Y, Muneta T, Tsuji K, Koga H, Sekiya I (2014). Mesenchymal stem cells in synovial fluid increase after meniscus injury. Clin Orthop Relat Res.

[30] De Schauwer C, Meyer E, Van De Walle GR, Van Soom A (2011). Markers of stemness in equine mesenchymal stem cells: a plea for uniformity. Theriogenology.

[31] Dominici M, Le Blanc K, Mueller I, Slaper-Cortenbach I, Marini F, Krause D (2006). Minimal criteria for defining multipotent mesenchymal stromal cells. The International Society for Cellular Therapy position statement. Cytotherapy.

[32] Vidal MA, Robinson SO, Lopez MJ, Paulsen DB, Borkhsenious O, Johnson JR (2008). Comparison of chondrogenic potential in equine mesenchymal stromal cells derived from adipose tissue and bone marrow. Vet Surg.

[33] Lovati AB, Corradetti B, Lange-Consiglio A, Recordati C, Bonacina E, Bizzaro D (2011). Comparison of equine bone marrow-, umbilical cord matrix and amniotic fluid-derived progenitor cells. Vet Res Commun.

[34] Kobayashi S, Takebe T, Inui M, Iwai S, Kan H, Zheng YW (2011). Reconstruction of human elastic cartilage by a CD44+ CD90+ stem cell in the ear perichondrium. Proc Natl Acad Sci USA.

[35] Corradeti B, Lange-Consiglio A, Barucca M, Cremonesi F, Bizzaro D (2011). Size-sieved subpopulations of mesenchymal stem cells from intervascular and perivascular equine umbilical cord matrix. Cell Prolif.

[36] Sakaguchi Y, Sekiya I, Yagishita K, Muneta T (2005). Comparison of human stem cells derived from various mesenchymal tissues: superiority of synovium as a cell source. Arthritis Rheum.

[37] Zhang S, Muneta T, Morito T, Mochizuki T, Sekiya I (2008). Autologous synovial fluid enhances migration of mesenchymal stem cells from synovium of osteoarthritis patients in tissue culture system. J Orthop Res.

[38] Sekiya I, Ojima M, Suzuki S, Yamaga M, Horie M, Koga H (2012). Human mesenchymal stem cells in synovial fluid increase in the knee with degenerated cartilage and osteoarthritis. J Orthop Res.

[39] Sun Y, Zheng Y, Liu W, Zheng Y, Zhang Z (2014). Synovium fragment-derived cells exhibit characteristics similar to those of dissociated multipotent cells in synovial fluid of the temporomandibular joint. PLoS One.

[40] Kitamura HP, Yanase H, Kitamura H, Iwanaga T (1999). Unique localization of protein gene product 9.5 in type B synoviocytes in the joints of the horse. J Histochem Cytochem.

[41] Mapp PI, Revell PA (1987). Ultrastructural localisation of muramidase in the human synovial membrane. Ann Rheum Dis.

[42] De Vita B, Campo LL, Listoni AJ, Maia L, Sudano MJ, Curcio BR (2013). Isolamento, caracterização e diferenciação de células-tronco mesenquimais do líquido amniótico equino obtido em diferentes idades gestacionais. Pesq Vet Bras.

[43] Pratheesh MD, Gade NE, Katiyar AN, Dubey PK, Sharma B, Saikumar G (2013). Isolation, culture and characterization of caprine mesenchymal stem cells derived from amniotic fluid. Res Vet Sci.

[44] Gao S, Zhao P, Lin C, Sun Y, Wang Y, Zhou Z (2014). Differentiation of human adipose-derived stem cells into neuron-like cells which are compatible. Tissue Eng Part A.

[45] Yoshimura H, Muneta T, Nimura A, Yokoyama A, Koga H, Sekiya I (2007). Comparison of rat mesenchymal stem cells derived from bone marrow, synovium, periosteum, adipose tissue, and muscle. Cell Tissue Res.

[46] Giovannini S, Brehm W, Mainil-Varlet P, Nesic D (2008). Multilineage differentiation potential of equine blood-derived fibroblast-like cells. Differentiation.

[47] Aggarwal S, Pittenger MF (2005). Human mesenchymal stem cells modulate allogeneic immune cell responses. Blood.

[48] Nauta AJ, Fibbe WE (2008). Review in translational hematology: immunomodulatory properties of mesenchymal stromal cells. Library.

[49] Peroni JF, Borjesson DL (2011). Anti-inflammatory and immunomodulatory activities of stem cells. Vet Clin North Am Equine Pract.

[50] van Lent PL, van den Berg WB (2013). Mesenchymal stem cell therapy in osteoarthritis: advanced tissue repair or intervention with smouldering synovial activation?. Arthritis Res Ther.

[51] Pezzanite LM, Fortier LA, Antczak DF, Cassano JM, Brosnahan MM, Miller D (2015). Equine allogeneic bone marrow-derived mesenchymal stromal cells elicit antibody responses in vivo. Stem Cell Res Ther.

